# Effects of Extra Virgin Olive Oil Polyphenols on Beta-Cell Function and Survival

**DOI:** 10.3390/plants10020286

**Published:** 2021-02-03

**Authors:** Nicola Marrano, Rosaria Spagnuolo, Giuseppina Biondi, Angelo Cignarelli, Sebastio Perrini, Leonardo Vincenti, Luigi Laviola, Francesco Giorgino, Annalisa Natalicchio

**Affiliations:** 1 Department of Emergency and Organ Transplantation, Section of Internal Medicine, Endocrinology, Andrology and Metabolic Diseases, University of Bari Aldo Moro, I-70124 Bari, Italy; nicola.marrano@uniba.it (N.M.); rosaria.spagnuolo06@gmail.com (R.S.); giuseppina.biondi2@gmail.com (G.B.); angelo.cignarelli@gmail.com (A.C.); sebastio.perrini@uniba.it (S.P.); luigi.laviola@uniba.it (L.L.); annalisa.natalicchio@uniba.it (A.N.); 2 Department of General Surgery, University Hospital Polyclinic, I-70124 Bari, Italy; dr.leonardo.vincenti@gmail.com

**Keywords:** extra virgin olive oil, phenolic compounds, diabetes, pancreatic beta-cells, insulin, apigenin

## Abstract

Extra virgin olive oil (EVOO) is a major component of the Mediterranean diet and is appreciated worldwide because of its nutritional benefits in metabolic diseases, including type 2 diabetes (T2D). EVOO contains significant amounts of secondary metabolites, such as phenolic compounds (PCs), that may positively influence the metabolic status. In this study, we investigated for the first time the effects of several PCs on beta-cell function and survival. To this aim, INS-1E cells were exposed to 10 μM of the main EVOO PCs for up to 24 h. Under these conditions, survival, insulin biosynthesis, glucose-stimulated insulin secretion (GSIS), and intracellular signaling activation (protein kinase B (AKT) and cAMP response element-binding protein (CREB)) were evaluated. Hydroxytyrosol, tyrosol, and apigenin augmented beta-cell proliferation and insulin biosynthesis, and apigenin and luteolin enhanced the GSIS. Conversely, vanillic acid and vanillin were pro-apoptotic for beta-cells, even if they increased the GSIS. In addition, oleuropein, p-coumaric, ferulic and sinapic acids significantly worsened the GSIS. Finally, a mixture of hydroxytyrosol, tyrosol, and apigenin promoted the GSIS in human pancreatic islets. Apigenin was the most effective compound and was also able to activate beneficial intracellular signaling. In conclusion, this study shows that hydroxytyrosol, tyrosol, and apigenin foster beta-cells’ health, suggesting that EVOO or supplements enriched with these compounds may improve insulin secretion and promote glycemic control in T2D patients.

## 1. Introduction

Diabetes mellitus describes a group of metabolic disorders characterized by chronically elevated glycemia. It represents one of the fastest-growing health challenges of the 21st century, with the number of adults living with diabetes having more than tripled over the past 20 years [1]. The International Diabetes Federation estimated 451 million (age 18–99 years) people with diabetes worldwide in 2017, with the estimation going up to 693 million for 2045 [1].

In its two main forms, diabetes is caused by immune-mediated beta-cell destruction (type 1 diabetes (T1D)) or by the loss of physiological beta-cell functional mass, often concomitant to reduced insulin sensitivity in peripheral insulin-dependent tissues (type 2 diabetes (T2D)).

The loss of beta-cell functional mass is a necessary and early condition in the development of T2D [2]. Accordingly, beta-cell restoration or regeneration should be strongly considered for the treatment, and possible cure, of T2D. Indeed, a truly efficient anti-diabetes therapeutic strategy capable of preventing the onset and progression of T2D should possess the capacity to stop beta-cell loss and/or promote the restoration of the fully functional beta-cell mass [2].

According to recommendations for the management of hyperglycemia in T2D from the American Diabetes Association and the European Association for the Study of Diabetes [3], lifestyle interventions, including the adoption of a healthy Mediterranean eating pattern, are effective and safe for improving glucose control in T2D and are recommended as first-line therapies from the time of diagnosis and as co-therapy for patients on glucose-lowering medications.

Extra virgin olive oil (EVOO) is a major component of the Mediterranean diet (MedDiet) and is appreciated worldwide because of its nutritional benefits in metabolic diseases, including T2D [4]. In the Prevención con Dieta Mediterránea (PREDIMED) study, a multicenter, randomized, parallel-group primary prevention trial conducted in Spain, participants were randomly assigned to receive MedDiet supplemented with EVOO, MedDiet supplemented with nuts, or a control low-fat diet, without interventions, to increase physical activity or lose weight [5,6]. After a median follow-up of 4.1 years, a statistically significant 40% relative risk reduction of new-onset T2D was observed in the group that received MedDiet supplemented with EVOO, but not in the group that received MedDiet supplemented with nuts, compared to the control diet [6], suggesting an important role of EVOO in diabetes prevention.

Traditionally, the high content of monounsaturated fatty acids (MUFAs), particularly oleic acid (C18:1, 55–83%), was considered to be responsible for the beneficial effects of EVOO [4]. Indeed, recent meta-analyses of randomized controlled trials have reported beneficial effects on metabolic parameters in T2D patients after replacing carbohydrates (~5–10% of total energy intake) with MUFAs [7,8,9]. It has been suggested, however, that most of the metabolic benefits of EVOO could be due to its minor components, particularly phenolic compounds (PCs) [4]. Accordingly, consuming EVOO rich in PCs (25 mL/day, 577 mg of PCs/kg) for a total of 4 weeks improved metabolic control in T2D patients compared to the consumption of refined olive oil with no PCs [10]. The phenolic content of EVOO consists of various phenolic classes, including phenolic acids (e.g., caffeic, vanillic, coumaric, ferulic, and sinapic acids), phenolic alcohols (e.g., tyrosol and hydroxytyrosol), secoiridoids (e.g., oleuropein), lignans (e.g., pinoresinol), and flavones (e.g., luteolin and apigenin) [11].

Although increasing data support the beneficial role of MedDiet and its components, especially PCs of EVOO, in T2D, the exact mechanisms responsible for these effects are not yet fully understood. Accordingly, in this study we investigated, for the first time, simultaneously but individually, the effects of the main EVOO PCs on beta-cell function and survival.

## 2. Results

### 2.1. Effects of EVOO PCs on Beta-Cell Survival

To evaluate the impact of PCs on beta-cell survival, INS-1E cells were treated for 24 h with 10 μM of the most important PCs identified in EVOO. To simplify the presentation of data, each compound was assigned an alphanumeric code (Table 1).

**Figure 1:**
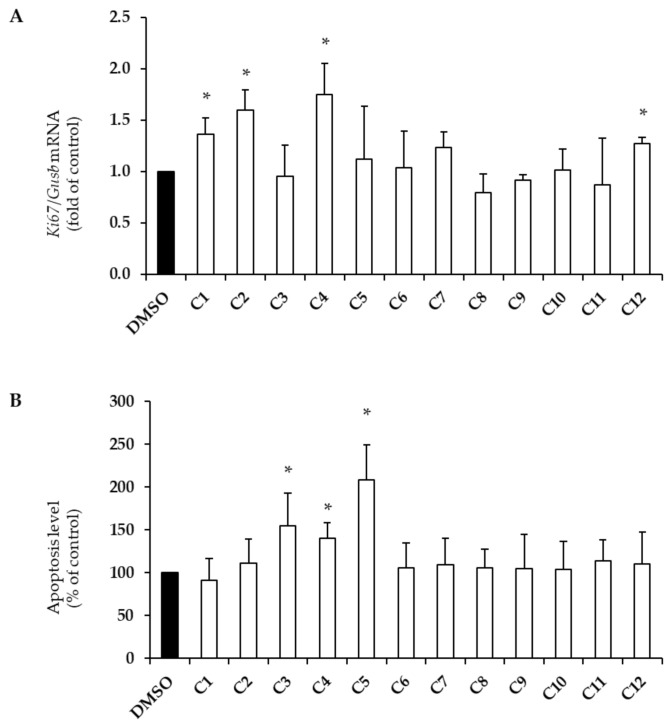
Effects of extra virgin olive oil (EVOO) phenolic compounds (PCs) (C1–C12, see Table 1) on beta-cell proliferation (**A**) and survival (**B**). INS-1E cells were treated for 24 h with 10 μM of each PC. Control cells were stimulated with DMSO only. (**A**) *Ki67* gene expression was evaluated by qRT-PCR analysis and normalized to *Gusb* gene expression. (**B**) Apoptosis was evaluated by measuring cytoplasmic oligonucleosomes with ELISA (data expressed as a percentage of controls). At least three independent experiments were performed. Data are expressed as means ± SD. Statistical significance was determined by one-way ANOVA (*p* < 0.05) followed by Bonferroni-corrected *t*-test (* *p* < 0.004 vs. DMSO). *Ki67*, a proliferation marker; *Gusb*, glucuronidase beta.

Beta-cell survival was assessed by measuring both mRNA levels of the marker of proliferation *Ki67* (Figure 1A) and apoptosis levels (Figure 1B).

Hydroxytyrosol (C1), tyrosol (C2), vanillic acid (C4), and apigenin (C12) significantly increased *Ki67* mRNA levels by approximatively 30–70% (Figure 1A; * *p* < 0.004 vs. DMSO). Surprisingly, caffeic acid (C3), vanillic acid (C4), and vanillin (C5) also increased apoptosis levels (1.5-, 1.4-, and 2-fold, respectively; Figure 1B; * *p* < 0.004 vs. DMSO).

### 2.2. Effects of EVOO PCs on Insulin Biosynthesis

**Figure 2:**
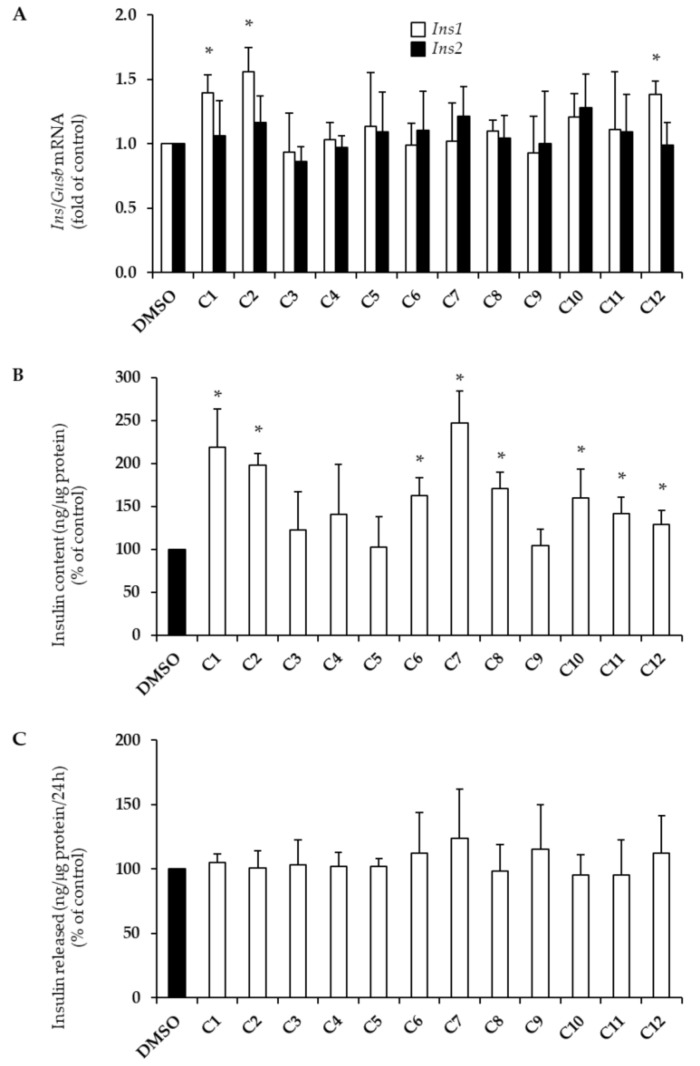
Effects of EVOO PCs (C1–C12, see Table 1) on insulin1 (*Ins1*, white bars) and insulin2 (*Ins2*, dark bars) mRNA levels (**A**), insulin content (**B**), and insulin release (**C**). INS-1E cells were treated for 24 h with 10 μM of each PC. Control cells were stimulated with DMSO only. (**A**) *Ins1* and *Ins2* gene expression was evaluated by qRT-PCR analysis and normalized to *Gusb* gene expression. (**B**) Insulin content was normalized to protein concentration and is expressed as a percentage of untreated controls. (**C**) Insulin release was measured in culture medium of INS-1E cells from (**B**), normalized to protein concentration and expressed as a percentage of untreated controls. At least three independent experiments were performed. Data are expressed as means ± SD. Statistical significance was determined by one-way ANOVA (*p* < 0.05) followed by Bonferroni-corrected *t*-test (* *p* < 0.004 vs. DMSO). *Ins1*, insulin1; *Ins2*, insulin2; *Gusb*, glucuronidase beta.

Hydroxytyrosol (C1), tyrosol (C2), and apigenin (C12) also increased insulin1 (*Ins1*) mRNA levels by approximatively 40–50% (Figure 2A; * *p* < 0.004 vs. DMSO) without inducing changes in insulin2 (*Ins2*) mRNA levels. Furthermore, hydroxytyrosol (C1), tyrosol (C2), *p*-coumaric acid (C6), ferulic acid (C7), sinapic acid (C8), (+)-pinoresinol (C10), oleuropein (C11), and apigenin (C12) significantly augmented insulin content levels to varying extents, ranging from 1.3- to 2.4-fold (Figure 2B; * *p* < 0.004 vs. DMSO). In addition, 24 h exposure to PCs did not induce any change in insulin release in the culture medium (Figure 2C).

### 2.3. Effects of EVOO PCs on Glucose-Stimulated Insulin Secretion

**Figure 3:**
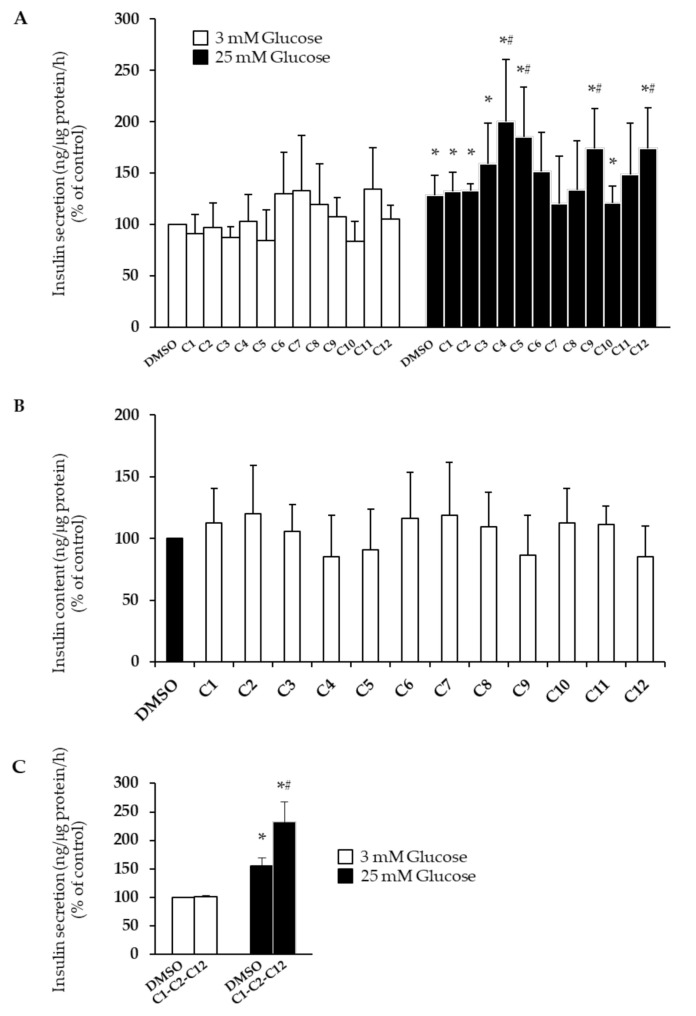
Effects of EVOO PCs (C1–C12, see Table 1) on the glucose-stimulated insulin secretion (GSIS) in INS-1E cells and human pancreatic islets. (**A**) INS-1E cells were treated for 1 h with 10 μM of each PC. Control cells were stimulated with DMSO only. (C) Human pancreatic islets were treated for 1 h with a mixture of 10 μM C1, 10 μM C2, and 10 μM C12. Control islets were stimulated with DMSO only. (**A**,**C**) GSIS was measured after 1 h at 3 mM glucose (white bars = basal secretion) followed by 1 h at 25 mM glucose (dark bars = stimulated secretion). Secretion was normalized to protein concentration and is expressed as a percentage of untreated controls. (**B**) Insulin content was measured in INS-1E cells from (**A**) after GSIS assay, normalized to protein concentration and expressed as a percentage of untreated controls. At least three independent experiments were performed. Data are expressed as means ± SD. Statistical significance was determined by one-way ANOVA (*p* < 0.05) followed by Bonferroni-corrected *t*-test (* *p* < 0.004 vs. 3 mM glucose; # *p* < 0.004 vs. DMSO for 3A; * *p* < 0.025 vs. 3 mM glucose; # *p* < 0.025 vs. DMSO for 3C).

For evaluation of glucose-stimulated insulin secretion (GSIS), INS-1E cells were treated for 1 h with 10 μM of each PC, cultured with 3 mM (basal) of glucose Krebs–Ringer bicarbonate HEPES buffer (KRBH) for 1 h and then cultured with 25 mM (stimulatory) of glucose KRBH for another hour. Interestingly, vanillic acid (C4), vanillin (C5), luteolin (C9), and apigenin (C12) enhanced the GSIS by ~1.9-, 1.4-, 1.3-, and 1.3-fold, respectively (Figure 3A; * *p* < 0.004 vs. 3 mM glucose; # *p* < 0.004 vs. DMSO). In contrast, p-coumaric acid (C6), ferulic acid (C7), sinapic acid (C8), and oleuropein (C11) reduced the ability of beta-cells to secrete insulin in response to stimulatory concentrations of glucose (Figure 3A). In addition, insulin content was measured in the same cells used for the GSIS assay (Figure 3B). Interestingly, unlike prolonged treatment (24 h, Figure 2B), short exposure to PCs (1 h) did not induce any changes in insulin content. Only a slight, not-significant reduction was observed in cells treated with vanillic acid (C4), vanillin (C5), luteolin (C9), and apigenin (C12), probably due to enhanced glucose-stimulated insulin release.

Furthermore, we evaluated the ability of a mixture of three PCs (i.e., 10 μM hydroxytyrosol (C1), 10 μM tyrosol (C2), and 10 μM apigenin (C12)) to promote the GSIS in human pancreatic islets. The components of the mixture were chosen for different reasons: while apigenin was the only PC to enhance all observed biological effects in INS-1E cells without influencing apoptosis, hydroxytyrosol and tyrosol, in addition to inducing beta-cell proliferation and insulin biosynthesis, are the only two PCs in EVOO to have already been included in a health claim by the European Food Safety Authority (EFSA) for their ability to protect blood lipids from oxidative stress [12]. Interestingly, we found that the mixture enhanced the GSIS by ~1.5-fold (Figure 3C; * *p* < 0.025 vs. 3 mM glucose; # *p* < 0.025 vs. DMSO).

### 2.4. Apigenin Activates Beta-Cell Intracellular Signaling

As mentioned above, apigenin was the only phenolic compound to enhance all observed biological effects without influencing apoptosis (Table 2).

**Figure 4:**
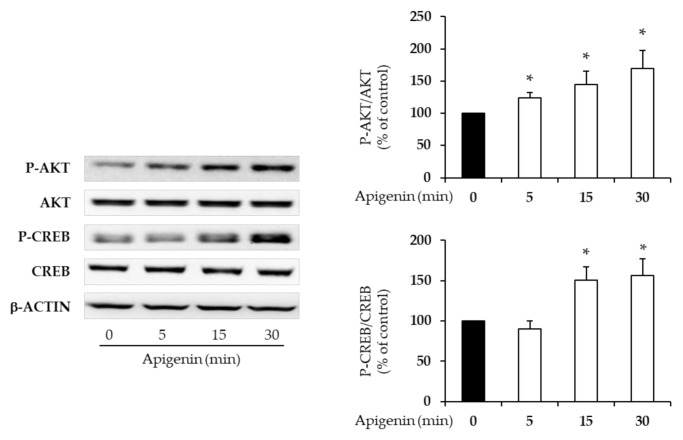
Effects of apigenin on beta-cell intracellular signaling. INS-1E cells were treated with 10 μM apigenin for 5, 15, and 30 min. Control cells were stimulated with DMSO only. Protein kinase B (AKT) and cAMP response element-binding protein (CREB) phosphorylation was measured by immunoblotting and quantified by densitometry. Densitometric analysis of the related bands was expressed as relative optical density, normalized using total AKT or CREB and expressed as a percentage of untreated controls. β-actin was used as a loading control. At least three independent experiments were performed. Data are expressed as means ± SD. Statistical significance was determined by one-way ANOVA (*p* < 0.05) followed by Bonferroni-corrected *t*-test (* *p* < 0.017 vs. no apigenin).

We, therefore, investigated whether apigenin is able to promote the phosphorylation/activation of protein kinase B (AKT) and cAMP response element-binding protein (CREB), which are key in the regulation of beta-cell mass and function [13,14]. Apigenin significantly induced both AKT and CREB phosphorylation, starting from 5 and 15 min of stimulation, respectively (Figure 4; * *p* < 0.017 vs. no apigenin).

## 3. Discussion

In this study, we investigated, for the first time, the effects of the main EVOO phenolic compounds on beta-cell function and survival. We found that hydroxytyrosol, tyrosol, and apigenin foster beta-cell health by promoting proliferation and improving insulin biosynthesis (hydroxytyrosol, tyrosol, and apigenin; Figure 1A and Figure 2), as well as by enhancing the GSIS (apigenin only; Figure 3A), without affecting apoptosis levels (Figure 1B). Interestingly, we also found that a mixture of hydroxytyrosol, tyrosol, and apigenin promotes the GSIS in human pancreatic islets (Figure 3C). Other PCs have shown beneficial properties in relation to single biological effects—proliferation (vanillic acid), insulin content (*p*-coumaric acid, ferulic acid, sinapic acid, (+)-pinoresinol, and oleuropein), and the GSIS (vanillic acid, vanillin, and luteolin). Surprisingly, several PCs also exerted detrimental effects. This was the case for caffeic acid, vanillic acid, and vanillin, which induced beta-cell apoptosis (Figure 1B), as well as *p*-coumaric acid, ferulic acid, sinapic acid, and oleuropein, which reduced the ability of beta-cells to secrete insulin in response to stimulatory concentrations of glucose (Figure 3A).

Recently, the ability of EVOO to improve metabolic control in T2D patients has been attributed to PCs, since the consumption of EVOO rich in PCs (25 mL/day, 577 mg of PCs/kg) reduced fasting plasma glucose, hemoglobin A1c (HbA1c) levels, and body mass index (BMI) in overweight T2D patients compared to the consumption of refined olive oil with no PCs [10]. The exact mechanisms responsible for these effects remain unclear, however.

Until now, only a few studies have examined the effects of EVOO PCs on beta-cell function and survival. Tyrosol was found to inhibit endoplasmic reticulum (ER)-induced beta-cell apoptosis [15], and caffeic acid enhanced the GSIS and glucose sensitivity in INS-1E cells [16]. Ferulic acid reportedly reduced beta-cell apoptosis in rats with streptozotocin (STZ)-induced diabetes [17] and prevented methylglyoxal-induced protein glycation, DNA damage, and apoptosis in pancreatic beta-cells [18]. Luteolin prevented cytokine- and uric-acid-induced pancreatic beta-cell dysfunction [19,20], likely by reducing ER stress [21], and oleuropein promoted insulin secretion and protected beta-cells from amyloid-, cytokine-, and H_2_O_2_-induced cytotoxicity [22,23,24,25]. Finally, apigenin attenuated pancreatic beta-cell damage in STZ-, 2-deoxy-D-ribose-, or cytokine-treated pancreatic beta-cells [20,26,27,28] through its protective effects on cellular antioxidant defense. To the best of our knowledge, our study is the first to evaluate simultaneously but individually the effects of the main EVOO PCs on both beta-cell function and survival.

Importantly, in our study, we used commercial standard PCs instead of PCs extracted directly from EVOO. This choice was driven by the need to reveal the specific effect of each individual PC on beta-cell function. In fact, while the ability of EVOO to improve metabolic control in T2D patients is already known [4,5,6], the further anti-diabetes properties of PC-enriched EVOO are still controversial [10,29]. Therefore, to identify both the ideal EVOO PCs mixture against diabetes and the valid criteria to evaluate the real anti-diabetes potential of a specific EVOO, it is crucial to understand whether and to what extent each PC contributes to these beneficial metabolic effects. For this purpose, the use of commercial standards has the advantage of being readily available in large quantities, of being not contaminated, and of ensuring reproducible data. On the other hand, PCs extracted directly from EVOO may have some disadvantages, such as (i) a low extraction yield, insufficient to perform experiments on cell lines; (ii) the retention of the solvents used for extraction or the mobile phases used for the separation, which could be cytotoxic; and (iii) difficulty in standardizing the extraction procedure and quantifying the real amount of extracted PCs [30]. Of course, the current study is not a point of arrival, while it is preparatory to further studies on PCs extracted directly from different varieties of EVOO, creating a knowledge base to appreciate a possible synergistic action of the PCs.

Relevant to this concept, it was recently demonstrated that the consumption of a diet rich in EVOO improves metabolic control and beta-cell survival and function in high-fat-diet-induced diabetes in mice; however, no additional beneficial effects were observed from EVOO containing higher levels of PCs [29]. According to our results, it is possible that the beneficial effects of some PCs (especially hydroxytyrosol, tyrosol, and apigenin) on beta-cell survival and function are counteracted by the detrimental effects of other PCs on apoptosis (caffeic acid, vanillic acid, and vanillin) and the GSIS (*p*-coumaric acid, ferulic acid, sinapic acid, and oleuropein). Indeed, we have shown that the same compound can exert both positive and negative effects. For example, vanillic acid and vanillin enhance the GSIS but increase apoptosis levels; similarly, *p*-coumaric acid, ferulic acid, sinapic acid, and oleuropein increase insulin content but reduce the GSIS. This could be due to the clonal heterogeneity of pancreatic beta-cells. In fact, it has recently emerged that different subpopulations of beta-cells may be differently targeted by insults or treatments, yielding potentially different responses in terms of function, proliferation, and survival [31].

Most studies agree that the beneficial effects of PCs are due to their antioxidant action. Here, we have also shown that apigenin, the only PC able to enhance all observed biological effects without influencing apoptosis, is able to phosphorylate and activate AKT and CREB (Figure 4), which are important positive regulators of beta-cell mass and function [13,14]. Previous studies have shown the ability of apigenin to activate AKT in numerous cellular systems [32]. Importantly, these protein intermediates are usually intracellular mediators of G-protein-coupled receptor or tyrosine kinase receptor signaling pathways. It could therefore be possible that apigenin acts through a specific receptor on the beta-cell surface. Further studies are required to shed light on the mechanisms of action through which PCs act on beta-cells.

Our study has some limitations. First, PCs undergo changes through intestinal and hepatic metabolism, in which they are hydrolyzed and later conjugated into their glucuronidated, methylated, or sulphated forms in order to be absorbed and become biologically active [33]. In our study, we tested only the effects of unmetabolized PCs, so we may have underestimated their biological effects. Second, in our study, the first effective dose of PCs on INS-1E cells was 10 μM. This concentration, although the lowest among those used in the literature, is apparently supra-physiological [34]. We have also tested lower doses of PCs without finding any biological effects (i.e., insulin secretion; data not shown). Since the ability to induce insulin secretion is a necessary condition for a compound to exert an anti-diabetes effect, we chose the dose of 10 μM regardless of the possible cytotoxicity that some PCs showed at that concentration (e.g., caffeic acid, vanillic acid, and vanillin). Phenolic concentration in EVOO ranges from 50 to 800 mg/kg, depending on variety, climate, area of growth, latitude, and ripeness of the olive [11]. In general, bioavailability studies in humans show that the absorption of olive oil phenols is likely larger than 55–66 mol% [35]. In particular, after ingestion of 30 mL of EVOO enriched with PCs, the maximum concentrations in plasma reached by hydroxytyrosol, tyrosol, and apigenin metabolites (hydroxytyrosol sulphate, tyrosol sulphate, and apigenin glucuronide) are approximatively 0.86, 0.95, and 0.09 μM, respectively [36], which are lower than the doses used in vitro. Nevertheless, it is difficult to predict the amount of PCs actually delivered to the beta-cells [2]. Third, more experiments on human beta-cells or human pancreatic islets, as well as targeted dietary intervention studies in animal models of T2D, are required to confirm our results, with the final goal of using specific EVOO PCs, alone or in combination, in preventive or therapeutic strategies against T2D.

In summary, this study shows that hydroxytyrosol, tyrosol, and apigenin, both alone and in combination, may preserve both function and survival of beta-cells, suggesting that EVOO or supplements enriched with these compounds may improve insulin secretion and promote glycemic control in T2D patients.

## 4. Materials and Methods

### 4.1. Cell Culture

Rat insulin-secreting INS-1E cells (passage 15–30; a kind gift from C. B. Wollheim, University of Geneva, Geneva, Switzerland) were grown in RPMI-1640 medium with 11.1 mM glucose supplemented with 10% fetal bovine serum (FBS), 100 IU/mL of penicillin, 100 μg/mL of streptomycin, and 1% nonessential amino acids (all from Thermo Fischer Scientific, Waltham, MA, USA). Cultures were kept in a monolayer at 37 °C in a humidified incubator gassed with 5% CO_2_. For experiments, cells were seeded in 6-well dishes up to 80% confluence.

### 4.2. Human Pancreatic Islets

Human pancreatic islets were isolated by collagenase digestion from pancreatic biopsies of non-obese, non-diabetic patients undergoing duodenocefalopancreasectomy (dpc) for Vater’s ampulla tumors. Biopsies were excised and processed with the approval of the regional ethics committee, and informed consent was obtained from each patient. Baseline characteristics of islets donors are reported in Table 3. Human pancreatic islets were cultured in Medium 199 with Earle’s salts (Sigma-Aldrich Inc., St. Louis, MO, USA) containing 5 mM glucose and supplemented with 10% FBS, 1% penicillin and streptomycin, 50 µg/mL of gentamicin (all from Thermo Fisher Scientific, Waltham, MA, USA), and 0.25 µg/mL of amphotericin (Aurogene s.r.l., Roma, Italy).

### 4.3. Chemicals and Treatments

Standards of hydroxytyrosol, tyrosol, caffeic acid, vanillic acid, vanillin, *p*-coumaric acid, ferulic acid, sinapic acid, luteolin, (+)-pinoresinol, oleuropein, and apigenin were all purchased from Sigma-Aldrich (St. Louis, MO, USA). Stock solutions of PCs were prepared in DMSO and stored at −20 °C. Cells were treated with 10 μM PCs, or a mixture thereof, for various times, as indicated. Under control conditions, cells were treated with DMSO only.

### 4.4. Immunoblotting and Measurement of Apoptosis

For protein analysis, cells were lysed in lysis buffer (50 mM HEPES pH 7.4, 1% Triton ×100, 150 mM NaCl, 1 mM MgCl_2_, 1 mM CaCl_2_, 10% glycerol, 10 mM NaPP, 10 mM NaF, and 4 mM EDTA) supplemented with protease and phosphatase inhibitors (Complete Mini Protease Inhibitor Cocktail Tablets and PhosStop Phosphatase Inhibitor Cocktail Tablets, Roche Diagnostic, Indianapolis, IN, USA). Protein concentration was determined using the Bradford assay (Biorad, Hercules, CA, USA). Equal amounts of protein (40 μg) were separated by SDS-PAGE and blotting on polyvinylidene fluoride (PVDF) membranes was performed using a Trans-Blot Turbo Transfer System (Biorad, Hercules, CA, USA). Membrane blocking and incubation with primary antibodies were performed with 5% skim milk in Tris-buffered saline with Tween, and then membranes were incubated with primary antibodies overnight at 4 °C, followed by washing with tris-buffered saline (TBS) and incubating with a horseradish-peroxidase-conjugated secondary antibody for 1 h at room temperature. Clarity Western ECL Substrate (Biorad, Hercules, CA, USA) was used for visualization of proteins with a Model 3000 VersadDoc Imaging System (Biorad, Hercules, CA, USA). The signal intensity of each protein band was then measured using Quantity One Software (Biorad, Hercules, CA, USA) and normalized to the corresponding total protein band. Anti-phosphorylated (p)AKT antibody (phosphorylation at Ser473 site; cat. no. 4060), anti-Akt antibody (4691), anti-pCREB antibody (phosphorylation at Ser133 site; 9198), and anti-CREB antibody (9197) were purchased from Cell Signaling Technology.

Apoptosis was measured using the Cell Death Detection ELISA^PLUS^ Kit (Roche Biochemicals, Indianapolis, IN, USA) according to the manufacturer’ instructions.

### 4.5. Gene Expression by Quantitative Reverse-Transcription Polymerase Chain Reaction (qRT-PCR)

Total RNA was isolated using the RNeasy Mini Kit (Qiagen, Hilden, Germany), with genomic DNA contamination eliminated via DNase digestion (Qiagen, Hilden, Germany). Total RNA (500 ng) was used as a template for cDNA synthesis using the High-Capacity cDNA Reverse Transcription Kit (Applied Biosystems, Weiterstadt, Germany). Primers were designed using Primer Express 3.0 (Applied Biosystems; see Table 4), and real-time PCR was carried out in a Biorad CFX Connect Real-Time System (Biorad, Hercules, CA, USA). Relative gene expression levels were determined by analyzing the changes in SYBR green fluorescence during PCR using the ΔΔCq method. The mRNA level of each gene was then normalized using *Gusb* (glucuronidase beta) as an internal control.

### 4.6. Measurement of Insulin Content and Glucose-Stimulated Insulin Secretion (GSIS)

To measure insulin content, INS-1E cells were lysed in non-denaturing lysis buffer (50 mM Tris HCl pH 7.4, 150 mM NaCl, 1 mM EDTA, and 1% Triton X-100, supplemented with protease and phosphatase inhibitors). Cell lysates were then cleared by centrifugation and frozen at −70 °C for subsequent determination of insulin concentrations (dilution factor 1:1000).

For GSIS assessment, INS-1E cells and human pancreatic islets were treated for 1 h with 10 μM PCs or a mixture of 10 μM C1, 10 μM C2, and 10 μM C12. Cells and islets were then cultured for 1 h in 3 mM glucose Krebs–Ringer bicarbonate HEPES buffer (KRBH; 136 mM NaCl, 4.7 mM KCl, 1.25 mM CaCl_2_, 1.25 mM MgCl_2_, 5 mMK H_2_PO_4_, 25 mM NaHCO_3_, 10 mM HEPES, and 0.5% BSA (pH 7.4)) in the presence of PCs and finally cultured for 1 h in 3 mM glucose KRBH buffer and for another hour in 25 mM glucose KRBH buffer. Supernatants were collected and frozen at −70 °C for subsequent determination of insulin concentrations (dilution factor 1:10).

Insulin concentration was assessed using a High-Range Rat Insulin ELISA kit (Mercodia AB, Sylveniusgatan, Uppsala, Sweden; detection range 3–150 μg/L; detection limit ≤1.5 µg/L).

### 4.7. Statistical Analysis

Data are expressed as means ± standard deviation (SD). At least three independent experiments were performed. Statistical significance was determined by one-way ANOVA (*p*-value < 0.05 was considered statistically significant) followed by Bonferroni-corrected paired *t*-test.

## Figures and Tables

**Table 1:** Each phenolic compound was assigned an alphanumeric code.

Compound	Code
Hydroxytyrosol	C1
Tyrosol	C2
Caffeic acid	C3
Vanillic acid	C4
Vanillin	C5
*p*-Coumaric acid	C6
Ferulic acid	C7
Sinapic acid	C8
Luteolin	C9
(+)-Pinoresinol	C10
Oleuropein	C11
Apigenin	C12

**Table 2:** Effects of EVOO PCs on the function and survival of INS-1E cells.

Compound	Proliferation	Apoptosis	Insulin1 mRNA Level	Insulin Content	GSIS
Hydroxytyrosol	↑	-	↑	↑	-
Tyrosol	↑	-	↑	↑	-
Caffeic acid	-	↑	-	-	-
Vanillic acid	↑	↑	-	-	↑
Vanillin	-	↑	-	-	↑
*p*-Coumaric acid	-	-	-	↑	↓
Ferulic acid	-	-	-	↑	↓
Sinapic acid	-	-	-	↑	↓
Luteolin	-	-	-	-	↑
(+)-Pinoresinol	-	-	-	↑	-
Oleuropein	-	-	-	↑	↓
Apigenin	↑	-	↑	↑	↑

↑, increased; ↓, reduced; -, no effect.

**Table 3:** Baseline characteristics of islet donors.

	Age (years)	Sex	BMI (kg/m^2^)	FPG (mg/dL)	Source
Islet preparation 1	66	F	19.38	82	dpc
Islet preparation 2	64	F	22.43	93	dpc
Islet preparation 3	61	M	18.38	82	dpc

BMI, body mass index; dpc, duodenocefalopancreasectomy; FPG, fasting plasma glucose.

**Table 4:** Primers used for qRT-PCR analysis.

Primer	Sequence (5′ → 3′)	Direction
rattus_ *Insulin1 (Ins1)*	CTGCCCAGGCTTTTGTCAA	Forward
rattus_ *Insulin1 (Ins1)*	TCCCCACACACCAGGTACAGA	Reverse
rattus_ *Insulin2 (Ins2)*	GCAAGCAGGTCATTGTTCCA	Forward
rattus_ *Insulin2 (Ins2)*	GGTGCTGTTTGACAAAAGCC	Reverse
rattus_ *Mki67*	GGACCCCAAAGAAGTGTTGA	Forward
rattus_ *Mki67*	GCTTCTCACCTGTTGCTTCC	Reverse
rattus_ *Gusb*	GACGTTGGGCTGGTGAACTAC	Forward
rattus_ *Gusb*	CACGGGCCACAATTTTGC	Reverse

## Data Availability

The data presented in this study are available in supplementary material here.

## Supplementary Materials

Supplementary Materials can be found at https://www.mdpi.com/2223-7747/10/2/286/s1.

## References

[1] Cho N., Shaw J., Karuranga S., Huang Y., Fernandes J.D.R., Ohlrogge A., Malanda B. (2018). IDF Diabetes Atlas: Global estimates of diabetes prevalence for 2017 and projections for 2045. Diabetes Res. Clin. Pr..

[2] Marrano N., Biondi G., Cignarelli A., Perrini S., Laviola L., Giorgino F., Natalicchio A. (2020). Functional loss of pancreatic islets in type 2 diabetes: How can we halt it?. Metabolism.

[3] Davies M.J., D’Alessio D.A., Fradkin J., Kernan W.N., Mathieu C., Mingrone G., Rossing P., Tsapas A., Wexler D.J., Buse J.B. (2018). Management of Hyperglycemia in Type 2 Diabetes, A Consensus Report by the American Diabetes Association (ADA) and the European Association for the Study of Diabetes (EASD). Diabetes Care.

[4] Mazzocchi A., Leone L., Agostoni C., Pali-Schöll I. (2019). The Secrets of the Mediterranean Diet. Does [Only] Olive Oil Matter?. Nutrients.

[5] Salas-Salvadó J., Bulló M., Babio N., Martínez-González M.Á., Ibarrola-Jurado N., Basora J., Estruch R., Covas M.I., Corella D., Arós F. (2010). Reduction in the Incidence of Type 2 Diabetes with the Mediterranean Diet: Results of the PREDIMED-Reus nutrition intervention randomized trial. Diabetes Care.

[6] Salas-Salvadó J., Bulló M., Estruch R., Ros E., Covas M.-I., Ibarrola-Jurado N., Corella D., Arós F., Gómez-Gracia E., Ruiz-Gutiérrez V. (2014). Prevention of Diabetes with Mediterranean Diets. Ann. Intern. Med..

[7] Qian F., Korat A.A., Malik V., Hu F.B. (2016). Metabolic Effects of Monounsaturated Fatty Acid–Enriched Diets Compared with Carbohydrate or Polyunsaturated Fatty Acid–Enriched Diets in Patients With Type 2 Diabetes: A Systematic Review and Meta-analysis of Randomized Controlled Trials. Diabetes Care.

[8] Schwingshackl L., Strasser B., Hoffmann G. (2011). Effects of Monounsaturated Fatty Acids on Cardiovascular Risk Factors: A Systematic Review and Meta-Analysis. Ann. Nutr. Metab..

[9] Schwingshackl L., Strasser B. (2012). High-MUFA Diets Reduce Fasting Glucose in Patients with Type 2 Diabetes. Ann. Nutr. Metab..

[10] Santangelo C., Filesi C., Varì R., Scazzocchio B., Filardi T., Fogliano V., D’Archivio M., Giovannini C., Lenzi A., Morano S. (2016). Consumption of extra-virgin olive oil rich in phenolic compounds improves metabolic control in patients with type 2 diabetes mellitus: A possible involvement of reduced levels of circulating visfatin. J. Endocrinol. Investig..

[11] Pedan V., Popp M., Rohn S., Nyfeler M., Bongartz A. (2019). Characterization of Phenolic Compounds and Their Contribution to Sensory Properties of Olive Oil. Molecules.

[12] EFSA Panel on Dietetic Products, Nutrition and Allergies (2011). Scientific Opinion on the substantiation of health claims related to olive oil and maintenance of normal blood LDL-cholesterol concentrations (ID 1316, 1332), maintenance of normal (fasting) blood concentrations of triglycerides (ID 1316, 1332), maintenan. EFSA J..

[13] Elghazi L., Rachdi L., Weiss A.J., Crasmeneur C., Bernalmizrachi E. (2007). Regulation of β-cell mass and function by the Akt/protein kinase B signalling pathway. Diabetes Obes. Metab..

[14] Dalle S., Quoyer J., Varin E., Costes S. (2011). Roles and Regulation of the Transcription Factor CREB in Pancreatic β-Cells. Curr. Mol. Pharmacol..

[15] Lee H., Im S.W., Jung C.H., Jang Y.J., Ha T.Y., Ahn J. (2016). Tyrosol, an olive oil polyphenol, inhibits ER stress-induced apoptosis in pancreatic β-cell through JNK signaling. Biochem. Biophys. Res. Commun..

[16] Bhattacharya S., Oksbjerg N., Young J.F., Jeppesen P.B. (2013). Caffeic acid, naringenin and quercetin enhance glucose-stimulated insulin secretion and glucose sensitivity in INS-1E cells. Diabetes Obes. Metab..

[17] Roy S., Metya S.K., Sannigrahi S., Rahaman N., Ahmed F. (2013). Treatment with ferulic acid to rats with streptozotocin-induced diabetes: Effects on oxidative stress, pro-inflammatory cytokines, and apoptosis in the pancreatic β cell. Endocrine.

[18] Sompong W., Cheng H., Adisakwattana S. (2016). Ferulic acid prevents methylglyoxal-induced protein glycation, DNA damage, and apoptosis in pancreatic β-cells. J. Physiol. Biochem..

[19] Ding Y., Shi X., Shuai X., Xu Y., Liu Y., Liang X., Wei D., Su D. (2014). Luteolin prevents uric acid-induced pancreatic b-cell dysfunction. J. Biomed. Res..

[20] Kim E.-K., Kwon K.-B., Song M.-Y., Han M.-J., Lee J.-H., Lee Y.-R., Lee J.-H., Ryu D.-G., Park B.-H., Park J.-W. (2007). Flavonoids Protect Against Cytokine-Induced Pancreatic β-Cell Damage Through Suppression of Nuclear Factor κB Activation. Pancreas.

[21] Wu W., He S., Shen Y., Zhang J., Wan Y., Tang X., Liu S., Yao X. (2020). Natural Product Luteolin Rescues THAP-Induced Pancreatic β-Cell Dysfunction through HNF4α Pathway. Am. J. Chin. Med..

[22] Chaari A. (2020). Inhibition of human islet amyloid polypeptide aggregation and cellular toxicity by oleuropein and derivatives from olive oil. Int. J. Biol. Macromol..

[23] Wu L., Velander P., Liu D., Xu B. (2017). Olive Component Oleuropein Promotes β-Cell Insulin Secretion and Protects β-Cells from Amylin Amyloid-Induced Cytotoxicity. Biochemistry.

[24] Cumaoğlu A., Ari N., Kartal M., Karasu Ç., Cumaoǧlu A. (2011). Polyphenolic Extracts from Olea europea L. Protect Against Cytokine-Induced β-Cell Damage Through Maintenance of Redox Homeostasis. Rejuvenation Res..

[25] Effects of Olive Leaf Polyphenols Against H₂O₂ Toxicity in Insulin Secreting β-Cells. https://pubmed.ncbi.nlm.nih.gov/21383995/.

[26] Wang N., Yi W.J., Tan L., Zhang J.H., Xu J., Chen Y., Qin M., Yu S., Guan J., Zhang R. (2017). Apigenin attenuates streptozotocin-induced pancreatic β cell damage by its protective effects on cellular antioxidant defense. Vitr. Cell. Dev. Biol. Anim..

[27] Esmaeili M.A., Zohari F., Sadeghi H. (2009). Antioxidant and Protective Effects of Major Flavonoids from Teucrium poliumon β-Cell Destruction in a Model of Streptozotocin-Induced Diabetes. Planta Med..

[28] Suh K.S., Oh S., Woo J.-T., Kim S.-W., Kim J.-W., Kim Y.S., Chon S. (2012). Apigenin attenuates 2-deoxy-D-ribose-induced oxidative cell damage in HIT-T15 pancreatic β-cells. Biol. Pharm. Bull..

[29] Jurado-Ruiz E., Álvarez-Amor L., Varela L.M., Berná G., Parra-Camacho M.S., Oliveras-Lopez M.J., Martínez-Force E., Rojas A., Hmadcha A., Soria B. (2019). Extra virgin olive oil diet intervention improves insulin resistance and islet performance in diet-induced diabetes in mice. Sci. Rep..

[30] Carrasco-Pancorbo A., Cerretani L., Bendini A., Segura-Carretero A., Gallina-Toschi T., Fernández-Gutiérrez A. (2005). Analytical determination of polyphenols in olive oils. J. Sep. Sci..

[31] Nasteska D., Hodson D.J. (2018). The role of beta cell heterogeneity in islet function and insulin release. J. Mol. Endocrinol..

[32] Salehi B., Venditti A., Sharifi-Rad M., Kręgiel D., Sharifi-Rad J., Durazzo A., Lucarini M., Santini A., Souto E.B., Novellino E. (2019). The Therapeutic Potential of Apigenin. Int. J. Mol. Sci..

[33] Manach C., Scalbert A., Morand C., Rémésy C., Jiménez L. (2004). Polyphenols: Food sources and bioavailability. Am. J. Clin. Nutr..

[34] Parkinson L., Cicerale S. (2016). The Health Benefiting Mechanisms of Virgin Olive Oil Phenolic Compounds. Molecules.

[35] Vissers M.N., Zock P.L., Katan M.B. (2004). Bioavailability and antioxidant effects of olive oil phenols in humans: A review. Eur. J. Clin. Nutr..

[36] Suárez M., Valls R.M., Romero M.-P., Macià A., Fernández S., Giralt M., Solà R., Motilva M.-J. (2011). Bioavailability of phenols from a phenol-enriched olive oil. Br. J. Nutr..

